# Crystal structure of *N*-hy­droxy­quinoline-2-carboxamide monohydrate

**DOI:** 10.1107/S2056989017005618

**Published:** 2017-04-28

**Authors:** Inna S. Safyanova, Kateryna A. Ohui, Iryna V. Omelchenko, Svitlana V. Shyshkina

**Affiliations:** a Department of Chemistry, National Taras Shevchenko University of Kyiv, Volodymyrska Street 64, 01601 Kiev, Ukraine; b SSI ‘Institute for Single Crystals’, National Academy of Sciences of Ukraine, 60 Nauki Ave., Kharkiv 61001, Ukraine; c V.N. Karazin Kharkiv National University, Department of Inorganic Chemistry, 4, Svobody Sq., Kharkiv 61001, Ukraine

**Keywords:** crystal structure, hydroxamic acids, hydrogen bonds, π–π stacking inter­actions

## Abstract

The title compound, C_10_H_8_N_2_O_2_·H_2_O, consists of an *N*-hy­droxy­quinoline-2-carboxamide mol­ecule in the keto tautomeric form and a water mol­ecule connected through an O—H⋯O hydrogen bond. The *N*-hy­droxy­quinoline-2-carboxamide mol­ecule has a nearly planar structure [maximum deviation = 0.062 (1) Å] and only the hy­droxy H atom deviates significantly from the mol­ecule plane. In the crystal, π–π stacking between the aromatic rings [inter­centroid distance = 3.887 (1) Å] and inter­molecular O—H⋯O hydrogen bonds organize the crystal components into columns extending along the *b*-axis direction.

## Abstract 2

The *N*-hy­droxy­quinoline-2-carboxamide mol­ecule has a nearly planar structure [maximum deviation = 0.062 (1) Å] and only the hy­droxy H atom deviates from the mol­ecule plane.

## Chemical context   

Hydroxamic acids are important bioligands that exhibit enzyme-inhibitory properties (Marmion *et al.*, 2013 ▸) and they have been studied extensively in coordination and bioinorganic chemistry (Ostrowska *et al.*, 2016 ▸; Golenya *et al.*, 2012*b* ▸; Świątek-Kozłowska *et al.*, 2000 ▸; Dobosz *et al.*, 1999 ▸). They are widely used in the preparation of metallacrowns (Golenya *et al.*, 2012*a* ▸; Gumienna-Kontecka *et al.*, 2013 ▸; Safyanova *et al.*, 2015 ▸) and as building blocks for synthesis of metal–organic frameworks and coordination polymers (Gumienna-Kontecka *et al.*, 2007 ▸; Golenya *et al.*, 2014 ▸; Pavlishchuk *et al.*, 2010 ▸, 2011 ▸).

*N*-Hy­droxy­quinoline-2-carboxamide, also known as quinoline-2-hydroxamic acid (QuinHA), has been used for the preparation of various metallacrown complexes (Stemmler *et al.*, 1999 ▸; Trivedi *et al.*, 2014 ▸; Jankolovits *et al.*, 2013 ▸). Presently, the Cambridge Structural Database (Groom *et al.*, 2016 ▸) contains ten entries on coordination compounds based on *N*-hy­droxy­quinoline-2-carboxamide, nine of which have been reported within the past four years.
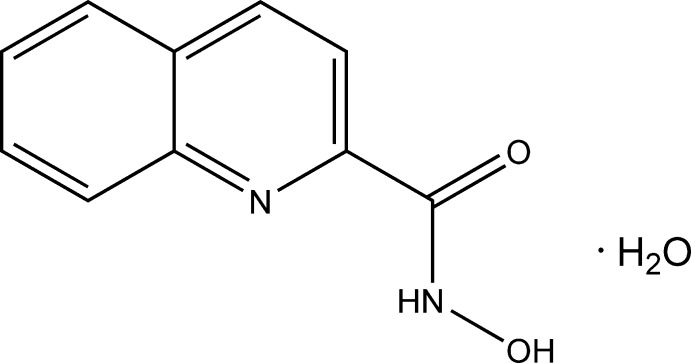


Structural information about the title compound is absent in the literature, however, and this will be useful in controlling the purity of the synthesized ligand and metal complexes by powder diffraction. It is well known that the products of such syntheses can be contaminated with impurities that result from hydrolysis or oxidation of the hydroxamic groups to the carb­oxy­lic group. In addition, syntheses of polynuclear complexes are often carried out with various metal-to-ligand ratios, so that in some cases an excessive qu­antity of the hydroxamic ligand can be present in the isolated samples.

## Structural commentary   

**Figure 1:**
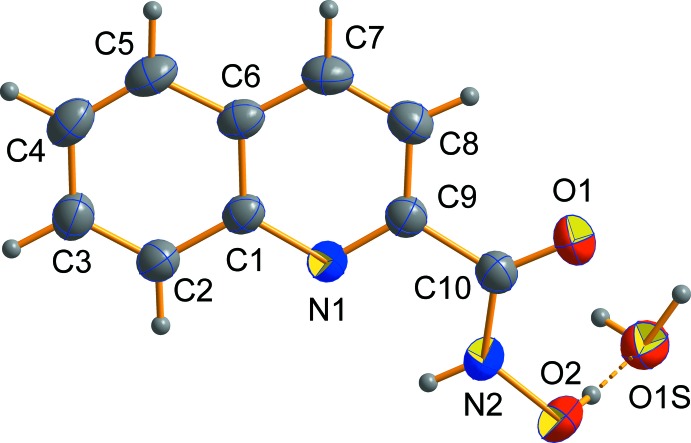
The asymmetric unit of the title compound, with displacement ellipsoids drawn at the 50% probability level. H atoms are shown as spheres of arbitrary radius. The dashed line indicates a hydrogen bond.

The mol­ecular structure of the title compound is presented in Fig. 1 ▸. It consists of an *N*-hy­droxy­quinoline-2-carboxamide mol­ecule in the keto tautomeric form {which is supported by the C=O [1.227 (2) Å] and C—N [1.317 (2) Å] bond lengths} and a water mol­ecule. The carbonyl group possesses a Z conformation against the N1 atom of the quinoline moiety and *E* conformation against the hy­droxy oxygen atom [torsion angles O2—N2—C10—O1 = 0.8 (2)° and N1—C9—C10—O1 −177.33 (14)°]. The *N*-hy­droxy­quinoline-2-carboxamide mol­ecule has an almost planar structure (non-hydrogen atoms are planar to within 0.03 Å). Only the H atom of the OH group deviates significantly from the mol­ecular plane: the C—N—O—H torsion angle of −75.1 (13)° is defined by the O—H⋯O hydrogen bond between hy­droxy group and the water mol­ecule. The C—N and C—C bond lengths in the quinoline moiety are typical for 2-substituted pyridine derivatives (Moroz *et al.*, 2012 ▸; Strotmeyer *et al.*, 2003 ▸; Krämer & Fritsky, 2000 ▸).

## Supra­molecular features   

**Figure 2:**
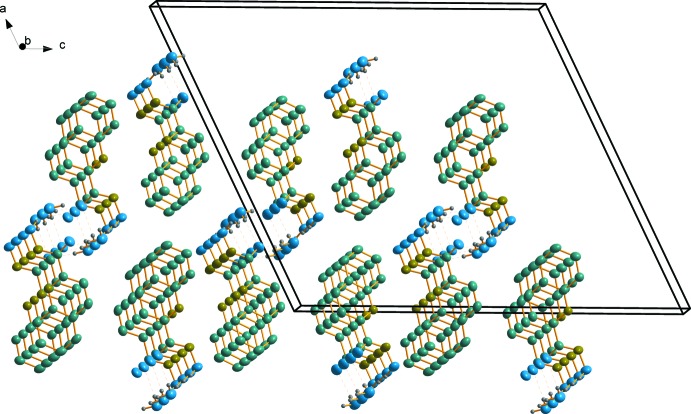
A packing diagram of the title compound. Hydrogen bonds (see Table 1 ▸) are indicated by dashed lines. H atoms not involved in hydrogen bonding have been omitted for clarity.

In the crystal, mol­ecules form columns along the *b* axis as a result of the π–π stacking inter­action between parallel quinoline moieties [symmetry operation *x*, *y* + 1, *z*; inter­planar separation 3.420 (1) Å, inter­centroid distance 3.887 (1) Å, displacement 1.846 (1) Å]. These columns are linked pairwise by the O—H⋯O hydrogen bonds (Table 1 ▸) *via* the bridging water mol­ecules (see Fig. 2 ▸). Each water mol­ecule forms two donor hydrogen bonds [H⋯O1 = 1.85 (2) and 2.15 (2) Å] with the carbonyl oxygen atom O1 and one acceptor hydrogen bond with the O—H group of the hydroxamic function that is the strongest hydrogen bond in the crystal [H⋯O2 = 1.67 (2) Å]. This latter hydrogen bond results in a shortened H⋯H contact between the water and hy­droxy hydrogen atoms [2.05 (3) Å]. The doubled columns are linked by weak N—H⋯π (2.71 Å, 159°) as well as van der Waals inter­actions. Weak inter­molecular C—H⋯O contacts (Table 1 ▸) are also observed in the crystal.

## Database survey   

A search of the Cambridge Structural Database (Groom *et al.*, 2016 ▸) reveals no crystal structures of isomeric *N*-hy­droxy­quinoline-carboxamides or their homologues. Two independent studies on the crystal structure of *N*-hy­droxy­picolinamide have been published recently (Chaiyaveij *et al.*, 2015 ▸; Safyanova *et al.*, 2016 ▸).

## Synthesis and crystallization   

The title compound was obtained by the reaction of a methanol solution of hy­droxy­amine with a mixture of quinaldic acid and ethyl chloro­formate in dry methyl­ene chloride in the presence of *N*-methyl­morpholine according to the reported procedure (Trivedi *et al.*, 2014 ▸). Light-yellow crystals suitable for X-ray diffraction were obtained from aqueous solution by slow evaporation at room temperature (yield 76%).

## Refinement   

Crystal data, data collection and structure refinement details are summarized in Table 2 ▸. All hydrogen atoms were found from the difference-Fourier maps and refined isotropically.

## Supplementary Material

Crystal structure: contains datablock(s) global, I. DOI: 10.1107/S2056989017005618/xu5902sup1.cif

Structure factors: contains datablock(s) I. DOI: 10.1107/S2056989017005618/xu5902Isup2.hkl

Supporting information file. DOI: 10.1107/S2056989017005618/xu5902Isup3.cml

CCDC reference: 1543825

Additional supporting information:  crystallographic information; 3D view; checkCIF report

## Figures and Tables

**Table 1:** Hydrogen-bond geometry (Å, °)

*D*—H⋯*A*	*D*—H	H⋯*A*	*D*⋯*A*	*D*—H⋯*A*
O1*S*—H1*SA*⋯O1^i^	0.85 (2)	2.15 (3)	2.9404 (19)	155 (2)
O1*S*—H1*SA*⋯O2^i^	0.85 (2)	2.54 (2)	3.0850 (18)	124 (2)
O1*S*—H1*SB*⋯O1^ii^	0.93 (2)	1.85 (2)	2.7783 (18)	176 (2)
O2—H2⋯O1*S*	0.97 (2)	1.67 (2)	2.6407 (18)	175 (2)
C3—H3⋯O2^iii^	0.999 (16)	2.518 (16)	3.493 (2)	165.0 (15)
C4—H4⋯O2^iv^	0.975 (19)	2.589 (18)	3.273 (2)	127.3 (12)
C5—H5⋯O1*S* ^v^	0.967 (17)	2.593 (17)	3.547 (2)	169.0 (13)

Symmetry codes: (i) 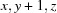; (ii) 
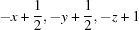
; (iii) 
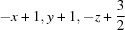
; (iv) 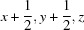; (v) 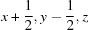.

**Table 2:** Experimental details

Crystal data	
Chemical formula	C_10_H_8_N_2_O_2_·H_2_O
*M*_r_	206.20
Crystal system, space group	Monoclinic, *C*2/*c*
Temperature (K)	298
*a*, *b*, *c* (Å)	21.613 (4), 3.8867 (4), 25.081 (5)
β (°)	115.37 (2)
*V* (Å^3^)	1903.7 (6)
*Z*	8
Radiation type	Mo *K*α
μ (mm^−1^)	0.11
Crystal size (mm)	0.4 × 0.1 × 0.1

Data collection	
Diffractometer	Agilent Xcalibur, Sapphire3
Absorption correction	Multi-scan (*CrysAlis PRO*; Agilent, 2014 ▸)
*T*_min_, *T*_max_	0.730, 1.000
No. of measured, independent and observed [*I* > 2σ(*I*)] reflections	7711, 2167, 1387
*R*_int_	0.040
(sin θ/λ)_max_ (Å^−1^)	0.650

Refinement	
*R*[*F* ^2^ > 2σ(*F* ^2^)], *wR*(*F* ^2^), *S*	0.040, 0.098, 0.97
No. of reflections	2167
No. of parameters	173
H-atom treatment	H atoms treated by a mixture of independent and constrained refinement
Δρ_max_, Δρ_min_ (e Å^−3^)	0.14, −0.17

Computer programs: *CrysAlis PRO* (Agilent, 2014 ▸), *SHELXS97* and *SHELXL97* (Sheldrick, 2008 ▸) and *OLEX2* (Dolomanov *et al.*, 2009 ▸).

## supplementary crystallographic information

### Crystal data

**Table:**

C_10_H_8_N_2_O_2_·H_2_O	*F*(000) = 864
*M**_r_* = 206.20	*D*_x_ = 1.439 Mg m^−^^3^
Monoclinic, *C*2/*c*	Mo *K*α radiation, λ = 0.71073 Å
*a* = 21.613 (4) Å	Cell parameters from 1341 reflections
*b* = 3.8867 (4) Å	θ = 3.8–27.4°
*c* = 25.081 (5) Å	µ = 0.11 mm^−^^1^
β = 115.37 (2)°	*T* = 298 K
*V* = 1903.7 (6) Å^3^	Needle, clear light yellow
*Z* = 8	0.4 × 0.1 × 0.1 mm

### Data collection

**Table:**

Agilent Xcalibur, Sapphire3 diffractometer	2167 independent reflections
Radiation source: Enhance (Mo) X-ray Source	1387 reflections with *I* > 2σ(*I*)
Graphite monochromator	*R*_int_ = 0.040
Detector resolution: 16.1827 pixels mm^-1^	θ_max_ = 27.5°, θ_min_ = 3.3°
ω scans	*h* = −28→28
Absorption correction: multi-scan (CrysAlis PRO; Agilent, 2014)	*k* = −5→4
*T*_min_ = 0.730, *T*_max_ = 1.000	*l* = −32→32
7711 measured reflections	

### Refinement

**Table:**

Refinement on *F*^2^	Primary atom site location: structure-invariant direct methods
Least-squares matrix: full	Secondary atom site location: difference Fourier map
*R*[*F*^2^ > 2σ(*F*^2^)] = 0.040	Hydrogen site location: inferred from neighbouring sites
*wR*(*F*^2^) = 0.098	H atoms treated by a mixture of independent and constrained refinement
*S* = 0.97	*w* = 1/[σ^2^(*F*_o_^2^) + (0.0454*P*)^2^] where *P* = (*F*_o_^2^ + 2*F*_c_^2^)/3
2167 reflections	(Δ/σ)_max_ < 0.001
173 parameters	Δρ_max_ = 0.14 e Å^−^^3^
0 restraints	Δρ_min_ = −0.17 e Å^−^^3^

### Special details

**Table:**

Geometry. All esds (except the esd in the dihedral angle between two l.s. planes) are estimated using the full covariance matrix. The cell esds are taken into account individually in the estimation of esds in distances, angles and torsion angles; correlations between esds in cell parameters are only used when they are defined by crystal symmetry. An approximate (isotropic) treatment of cell esds is used for estimating esds involving l.s. planes.
Refinement. Refinement of F^2^ against ALL reflections. The weighted R-factor wR and goodness of fit S are based on F^2^, conventional R-factors R are based on F, with F set to zero for negative F^2^. The threshold expression of F^2^ > 2sigma(F^2^) is used only for calculating R-factors(gt) etc. and is not relevant to the choice of reflections for refinement. R-factors based on F^2^ are statistically about twice as large as those based on F, and R- factors based on ALL data will be even larger.

### Fractional atomic coordinates and isotropic or equivalent isotropic displacement parameters (Å^2^)

**Table:**

	*x*	*y*	*z*	*U*_iso_*/*U*_eq_	
O1	0.32060 (5)	0.1130 (3)	0.55333 (5)	0.0582 (3)	
O2	0.27884 (5)	0.3220 (3)	0.63530 (5)	0.0604 (4)	
N1	0.46916 (5)	0.5267 (3)	0.65622 (5)	0.0381 (3)	
N2	0.34314 (6)	0.3877 (4)	0.63808 (6)	0.0523 (4)	
H2A	0.3705 (8)	0.508 (4)	0.6691 (8)	0.060 (5)*	
H2	0.2492 (10)	0.479 (5)	0.6052 (10)	0.090*	
H1SA	0.2274 (11)	0.899 (6)	0.5506 (10)	0.090*	
H1SB	0.1922 (10)	0.628 (5)	0.5141 (10)	0.090*	
C1	0.53537 (6)	0.6016 (3)	0.66788 (6)	0.0359 (3)	
C2	0.57630 (7)	0.7759 (4)	0.72050 (7)	0.0424 (4)	
H2B	0.5567 (7)	0.834 (4)	0.7473 (7)	0.050 (4)*	
C3	0.64285 (8)	0.8497 (4)	0.73422 (8)	0.0469 (4)	
H3	0.6734 (8)	0.966 (4)	0.7719 (7)	0.060 (5)*	
C4	0.67118 (8)	0.7562 (4)	0.69502 (8)	0.0491 (4)	
H4	0.7190 (8)	0.810 (4)	0.7053 (7)	0.055 (4)*	
C5	0.63321 (8)	0.5923 (4)	0.64395 (8)	0.0476 (4)	
H5	0.6504 (8)	0.527 (4)	0.6156 (7)	0.059 (5)*	
C6	0.56373 (7)	0.5090 (3)	0.62831 (7)	0.0391 (3)	
C7	0.52127 (8)	0.3386 (4)	0.57617 (7)	0.0461 (4)	
H7	0.5385 (8)	0.275 (4)	0.5474 (7)	0.058 (5)*	
C8	0.45526 (8)	0.2675 (4)	0.56466 (7)	0.0453 (4)	
H8	0.4245 (8)	0.161 (4)	0.5306 (7)	0.054 (5)*	
C9	0.43182 (7)	0.3633 (3)	0.60679 (6)	0.0380 (3)	
C10	0.35991 (7)	0.2754 (4)	0.59651 (7)	0.0409 (4)	
O1S	0.20068 (6)	0.7378 (3)	0.54961 (6)	0.0609 (4)	

### Atomic displacement parameters (Å^2^)

**Table:**

	*U*^11^	*U*^22^	*U*^33^	*U*^12^	*U*^13^	*U*^23^
O1	0.0489 (6)	0.0750 (8)	0.0461 (7)	−0.0163 (5)	0.0158 (6)	−0.0158 (6)
O2	0.0396 (6)	0.0884 (9)	0.0583 (8)	−0.0119 (5)	0.0259 (6)	0.0006 (6)
N1	0.0342 (6)	0.0446 (7)	0.0360 (7)	−0.0008 (5)	0.0153 (5)	0.0006 (6)
N2	0.0363 (7)	0.0751 (10)	0.0480 (9)	−0.0148 (7)	0.0206 (7)	−0.0130 (8)
C1	0.0326 (7)	0.0384 (7)	0.0380 (8)	0.0029 (6)	0.0163 (6)	0.0065 (6)
C2	0.0378 (8)	0.0501 (9)	0.0412 (9)	−0.0006 (7)	0.0187 (7)	0.0014 (7)
C3	0.0376 (8)	0.0518 (9)	0.0488 (11)	−0.0046 (7)	0.0162 (8)	0.0013 (8)
C4	0.0363 (8)	0.0525 (10)	0.0616 (12)	0.0015 (7)	0.0240 (8)	0.0078 (8)
C5	0.0442 (9)	0.0510 (9)	0.0588 (11)	0.0057 (7)	0.0328 (9)	0.0065 (8)
C6	0.0395 (7)	0.0395 (8)	0.0430 (9)	0.0062 (6)	0.0221 (7)	0.0062 (7)
C7	0.0522 (9)	0.0505 (9)	0.0438 (10)	0.0055 (7)	0.0282 (8)	0.0008 (7)
C8	0.0481 (9)	0.0490 (9)	0.0385 (9)	−0.0015 (7)	0.0182 (8)	−0.0058 (7)
C9	0.0374 (7)	0.0387 (7)	0.0371 (9)	0.0003 (6)	0.0154 (7)	0.0025 (6)
C10	0.0391 (8)	0.0453 (8)	0.0357 (9)	−0.0023 (6)	0.0135 (7)	0.0020 (7)
O1S	0.0549 (7)	0.0724 (8)	0.0607 (8)	−0.0107 (6)	0.0298 (7)	−0.0151 (7)

### Geometric parameters (Å, º)

**Table:**

O1—C10	1.2266 (17)	C4—H4	0.974 (16)
O2—N2	1.3851 (15)	C4—C5	1.349 (2)
O2—H2	0.97 (2)	C5—H5	0.966 (16)
N1—C1	1.3635 (17)	C5—C6	1.417 (2)
N1—C9	1.3169 (17)	C6—C7	1.401 (2)
N2—H2A	0.884 (18)	C7—H7	0.976 (17)
N2—C10	1.316 (2)	C7—C8	1.357 (2)
C1—C2	1.408 (2)	C8—H8	0.927 (16)
C1—C6	1.4183 (19)	C8—C9	1.404 (2)
C2—H2B	0.962 (16)	C9—C10	1.5017 (19)
C2—C3	1.358 (2)	O1S—H1SA	0.84 (2)
C3—H3	0.999 (17)	O1S—H1SB	0.93 (2)
C3—C4	1.410 (2)		
			
N2—O2—H2	103.8 (12)	C4—C5—C6	120.80 (15)
C9—N1—C1	117.93 (12)	C6—C5—H5	115.6 (10)
O2—N2—H2A	114.9 (11)	C5—C6—C1	118.21 (14)
C10—N2—O2	120.66 (14)	C7—C6—C1	117.81 (13)
C10—N2—H2A	124.5 (11)	C7—C6—C5	123.98 (14)
N1—C1—C2	118.95 (13)	C6—C7—H7	120.4 (9)
N1—C1—C6	121.61 (13)	C8—C7—C6	120.22 (14)
C2—C1—C6	119.44 (13)	C8—C7—H7	119.4 (9)
C1—C2—H2B	118.7 (9)	C7—C8—H8	124.1 (10)
C3—C2—C1	120.73 (15)	C7—C8—C9	118.11 (15)
C3—C2—H2B	120.6 (9)	C9—C8—H8	117.8 (10)
C2—C3—H3	122.3 (9)	N1—C9—C8	124.30 (13)
C2—C3—C4	119.85 (16)	N1—C9—C10	116.29 (12)
C4—C3—H3	117.8 (9)	C8—C9—C10	119.41 (14)
C3—C4—H4	119.2 (9)	O1—C10—N2	123.24 (14)
C5—C4—C3	120.96 (15)	O1—C10—C9	122.90 (13)
C5—C4—H4	119.8 (9)	N2—C10—C9	113.86 (13)
C4—C5—H5	123.6 (10)	H1SA—O1S—H1SB	102.7 (19)
			
O2—N2—C10—O1	0.8 (2)	C2—C3—C4—C5	−0.3 (2)
O2—N2—C10—C9	−178.96 (12)	C3—C4—C5—C6	−0.1 (2)
N1—C1—C2—C3	178.81 (13)	C4—C5—C6—C1	−0.2 (2)
N1—C1—C6—C5	−179.24 (12)	C4—C5—C6—C7	−179.86 (15)
N1—C1—C6—C7	0.4 (2)	C5—C6—C7—C8	179.76 (14)
N1—C9—C10—O1	−177.33 (14)	C6—C1—C2—C3	−1.3 (2)
N1—C9—C10—N2	2.48 (19)	C6—C7—C8—C9	−1.2 (2)
C1—N1—C9—C8	−1.4 (2)	C7—C8—C9—N1	1.9 (2)
C1—N1—C9—C10	178.08 (11)	C7—C8—C9—C10	−177.53 (13)
C1—C2—C3—C4	1.0 (2)	C8—C9—C10—O1	2.2 (2)
C1—C6—C7—C8	0.1 (2)	C8—C9—C10—N2	−178.00 (14)
C2—C1—C6—C5	0.91 (19)	C9—N1—C1—C2	−179.94 (12)
C2—C1—C6—C7	−179.44 (13)	C9—N1—C1—C6	0.20 (19)

### Hydrogen-bond geometry (Å, º)

**Table:**

*D*—H···*A*	*D*—H	H···*A*	*D*···*A*	*D*—H···*A*
O1*S*—H1*SA*···O1^i^	0.85 (2)	2.15 (3)	2.9404 (19)	155 (2)
O1*S*—H1*SA*···O2^i^	0.85 (2)	2.54 (2)	3.0850 (18)	124 (2)
O1*S*—H1*SB*···O1^ii^	0.93 (2)	1.85 (2)	2.7783 (18)	176 (2)
O2—H2···O1*S*	0.97 (2)	1.67 (2)	2.6407 (18)	175 (2)
C3—H3···O2^iii^	0.999 (16)	2.518 (16)	3.493 (2)	165.0 (15)
C4—H4···O2^iv^	0.975 (19)	2.589 (18)	3.273 (2)	127.3 (12)
C5—H5···O1*S*^v^	0.967 (17)	2.593 (17)	3.547 (2)	169.0 (13)

Symmetry codes: (i) *x*, *y*+1, *z*; (ii) −*x*+1/2, −*y*+1/2, −*z*+1; (iii) −*x*+1, *y*+1, −*z*+3/2; (iv) *x*+1/2, *y*+1/2, *z*; (v) *x*+1/2, *y*−1/2, *z*.
